# Reductive Effect of Acitretin on Blood Glucose Levels in Chinese Patients With Psoriasis

**DOI:** 10.3389/fmed.2021.764216

**Published:** 2021-12-16

**Authors:** Hua Qian, Yehong Kuang, Juan Su, Menglin Chen, Xiang Chen, Chengzhi Lv, Wangqing Chen, Wu Zhu

**Affiliations:** ^1^Department of Dermatology, Hunan Engineering Research Center of Skin Health and Disease, Hunan Key Laboratory of Skin Cancer and Psoriasis, Xiangya Hospital, Central South University, Changsha, China; ^2^National Clinical Research Center for Geriatric Disorders, Xiangya Hospital, Central South University, Changsha, China; ^3^Department of Dermatology, Soochow University Affiliated Children's Hospital, Suzhou, China; ^4^Department of Dermatology, Dalian Dermatology Hospital, Dalian, China

**Keywords:** psoriasis, acitretin, glucose, lipids, metabolism

## Abstract

**Background:** Psoriasis is a skin condition associated with increased risks of developing metabolic diseases, such as diabetes and hyperlipidaemia. Retinoid drugs, including acitretin, are commonly used to treat psoriasis due to its low cost and tolerable side effects.

**Objective:** This study aimed to explore the influence of acitretin on patients' metabolism levels, especially lipid and glucose.

**Methods:** In this retrospective study, a total of 685 psoriatic patients and 395 age/sex matched controls were enrolled. The demographic and biochemical indexes of each participant were recorded. Acitretin (30 mg/d) combined with the topical ointment calcipotriol was used to treat the psoriatic patients, and the glucose and lipid profiles of patients before and after acitretin treatment were analyzed.

**Results:** The blood glucose levels of 685 psoriasis patients were significantly higher than that of the control group (*P* < 0.001), while the blood lipid levels showed no difference between psoriatic patients and the matched controls. Triglyceride and low-density lipoprotein levels were significantly increased in 247 patients (*P* < 0.05) after 8 weeks of treatment with acitretin. Interestingly, there was a remarkable downward trend in body mass index (BMI) and blood glucose levels (*P* < 0.05) after acitretin treatment. Additionally, expression of both *GLUT1* and *GLUT4* in HaCaT and HepG2 cells were significantly increased when treated with acitretin. Compared to acitretin-free cells, the uptake of 2-NBDG was significantly higher in HaCaT and HepG2 cells after incubation with 5000 ng/mL acitretin for 36 h.

**Conclusion:** Acitretin plays a significant role of reducing the blood glucose level in psoriasis patients. The mechanism of lowering blood glucose may be through increasing glucose intake by cells, thereby reducing glucose levels in the peripheral blood.

## Background

Psoriasis, an incurable disease caused by both genetic and environmental factors, affects about 2–3% of the world's population, resulting in serious economic burdens (1). Psoriasis mainly manifests as an abnormal proliferation and differentiation of keratinocytes, as well as an abnormal infiltration and imbalance of inflammatory cells (2). In addition to affecting the skin, multiple studies have shown a correlation between psoriasis and elevated blood sugar and blood lipid levels (3, 4). It has been reported that genes, such as *GLUT1, GLUT2, GLUT4, GCK4*, and *INSIG1*, are commonly associated with glucose uptake, transport, and metabolism. Studies have also revealed that psoriatic patients are at an increased risk for cardiovascular diseases, obesity, diabetes, hypertension, dyslipidaemia, and other comorbidities when compared with non-psoriatic individuals (2, 5–7). There are several classes of drugs used to treat psoriasis including retinol derivatives, methotrexate (MTX), and biologicals. As a non-immunosuppressant, retinol derivatives are widely used in Chinese patients with psoriasis.

Previous studies have shown that vitamin A plays an important role in human development and metabolism. After modification of its chemical structure, scientists have obtained numerous derivatives of vitamin A, known as retinoids. Previous studies have reported that retinoids can promote differentiation of keratinocytes and inhibit its proliferation, thus enhancing the skin tissue immunity, and alleviating inflammation (8–10). Acitretin, a retinoid drug, is widely used for the treatment of pustular psoriasis and moderate to severe plaque psoriasis in China (11–13). Compared to the parent drug etretinate, with a long half-life 120 days, acitretin has a shorter half-life (50–60 h), making it a potentially safer alternative (14, 15). Studies involving acitretin treatment revealed that with low dosage (<50 mg/d), ~23–52% of patients achieved a 75% improvement compared to the baseline psoriasis area and severity index (PASI) value (PASI75), and 66–85% of the patients achieved a PASI50 improvement (8, 14). In contrast, a higher dosage (~50–75 mg/d) of acitretin resulted in an increase in the efficacy and a decrease in the side effects (8). The most common adverse drug reaction (ADR) of acitretin is dyslipidaemia, which increases the risk of atherosclerosis in patients treated with retinoids over a long period of time (16). With the reported ADR of acitretin, and the correlation between psoriasis and metabolic changes, this study was designed to examine the glucose and lipid metabolic profile of Chinese patients with psoriasis after the treatment of acitretin.

## Methods and Patients

### Patients

The demographic and blood tests information of 685 patients with psoriasis, who visited the dermatology department, Xiangya Hospital of Central South University, from 2014 to 2020, were retrospectively analyzed. The study was approved by the Ethics Committee of Xiangya Hospital, with the clinical trial registration number ChiCTR-OCH-14004518 (Chinese Clinical Trial Registry online, https://www.chictr.org.cn/showproj.aspx?proj=5054). The study was conducted in accordance with the Declaration of Helsinki, and informed written consents were obtained from every patient. All the patients were diagnosed with plaque psoriasis by at least two clinicians. For the control group, 395 age- and gender-matched individuals with no prior clinical diagnosis of psoriasis, were recruited in the physical examination center of Xiangya Hospital. Blood tests, liver and kidney functions, blood glucose level, and other indicators were recorded in both controls and psoriatic patients before and after the treatment.

### Treatment Regimens

Among the 685 psoriatic patients, 247 patients received acitretin 30 mg/d treatment in combination with the topical ointment calcipotriol for 8–16 weeks; Acitretin was obtained from Chongqing Huapont Pharmaceutical Co., Ltd., China; Calcipotriol was obtained from Bright Future Pharm, Hongkong.

### Cell Culture and Acitretin Incubation

Both HepG2 and HaCaT cells were stored in our laboratory at −80°C, and were culturedat 37°C in a humidified 5% CO_2_ atmosphere in Dulbecco's modified Eagle's medium (DMEM) (Hyclone Laboratories Inc., Logan, UT, USA) supplemented with 10% foetal bovine serum (FBS; Biological Industries, Israel), 100 mg/mL penicillin, and 100 mg/mL streptomycin (Invitrogen, Carlsbad, CA, USA). Cell passage was carried out in a 1:3 proportion every 3–5 days. Acitretin 5000 ng/mL was then used to incubate HepG2 and HaCaT cells for 36 h.

### Detecting the Uptake of 2-deoxy-2-[(7-nitro-2,1,3-benzoxadiazol-4-yl)amino]-β-Dglucopyranose (2-NBDG)

2-NBDG (MKBio, Shanghai, China, CAS NO: 186689-07-6), a green, fluorescent deoxyglucose analog, was used to monitor the cell glucose uptake using flow cytometry or fluorescence microscopy, as an indicator of cellular viability (17). To detect the uptake of 2-NBDG, 1 × 10^5^ cells/well were seeded in a 12-well plate, six of which were incubated with complete DMEM as the control, and the remaining six wells incubated with DMEM and acitretin 5000 ng/mL as the acitretin group. After 36 h, the cells were washed three times with 200 μL cold phosphate-buffered saline (PBS), treated with 0.5 mM 2-NBDG for 30 min, then washed thrice with cold PBS. This process was repeated three times.

The uptake of 2-NBDG by both HaCaT and HepG2 cells was observed by fluorescence microscopy (Nikon Eclipse TS2R, Japan). The cells were then resuspended with 200 μL PBS per well and assessed using the BD Aucrri C6 flow cytometer, as previously described (17).

### RNA Extraction and Real Time PCR Analysis

The total RNA of the cells was extracted and 2 μg RNA was reverse transcribed into cDNA per manufacturer's instructions (Vazyme Biotech Co., Ltd, China). The real time PCR primers of human genes, *GLUT1, GLUT2, GLUT4, GCK4*, and *INSIG1*, are shown in Supplementary Table 1; Taq DNA polymerase (Continental Lab Products, San Diego, CA, USA) was used according to the manufacturer's instructions. Quantitative real time PCR was carried out on a Real Time PCR Detection System, Applied Biosystems 7500 (Applied Biosystems, Waltham, MA, USA). The PCR analyses were performed in 40 cycles with the following parameters: 94°C, 45 s; 60°C, 45 s.

### Statistical Methods

The clinical data and laboratory parameters of all the enrolled participants were collated and analyzed. Continuous variables (e.g., age, BMI, and blood glucose and blood lipid levels) were expressed as the mean ± standard deviation (SD). An independent sample *t*-test was used to analyse whether the distribution of continuous variables had statistical differences between psoriatic patients and controls. Categorical variables were mainly expressed in terms of frequency (or percentage), and the Chi-squared test was used to compare the distribution between the different groups. A paired sample *t*-test was used to examine changes in BMI and blood glucose and lipid levels before and after treatment. Statistical significance was set at *P* < 0.05.

## Results

### Blood Glucose and Lipid Levels in Psoriatic Patients and Controls

In this study, we enrolled 685 patients with psoriasis and 395 age/gender-matched controls. There were no observed differences in the BMI values between these two groups (23.34 ± 5.16 vs. 23.07 ± 4.25 kg/m^2^, *P* = 0.353). As mentioned above, studies have shown that fasting blood glucose and lipid levels are significantly higher in psoriatic patients than those without psoriasis. We confirmed that the 685 patients with psoriasis had higher blood glucose levels than the controls (5.76 ± 1.31 vs. 5.38 ± 1.14 mmol/L, *P* < 0.001). However, the blood lipid levels showed no difference between the psoriatic patients and the controls (*P* > 0.05), as shown in Table 1, and the other laboratory parameters of the two groups are also shown in Supplementary Table 2.

**Table 1:** Fasting blood glucose and lipids levels in patients with psoriasis and control groups.

**Laboratory tests parameters**	**Controls** **(*N* = 395)**	**Psoriasis** **(*N* = 685)**	**Sig**.	**OR [95% CI]**
Gender ratio (Male vs. Female)	66.48% vs. 33.52%	68.18% vs. 31.82%	0.501	NA
Age (year)	42.15 ± 12.05	41.94 ± 13.12	0.794	NA
BMI (kg/m^2^)	23.34 ± 5.16	23.07 ± 4.25	0.353	NA
Blood glucose (mmol/L)	5.38 ± 1.14	5.76 ± 1.31	<0.001***	NA
Triglycerides (TG) (mmol/L)	1.62 ± 1.67	1.65 ± 1.48	0.748	NA
Total Cholesterol (TC) (mmol/L)	4.90 ± 1.02	4.90 ± 1.05	0.954	NA
High density lipoprotein (HDL) (mmol/L)	1.48 ± 0.34	1.44 ± 0.4	0.103	NA
Low density lipoprotein (LDL) (mmol/L)	2.75 ± 0.84	2.82 ± 0.84	0.194	NA
HDL/TC	0.39 ± 1.55	0.30 ± 0.13	0.159	NA
Abnormal blood glucose (>5.6 mmol/L)	33(8.35%)	142(20.74%)	<0.001***	2.871 [1.868, 4.412]
Abnormal triglycerides (>1.70 mmol/L)	114(28.93%)	192(28.07%)	0.766	0.958 [0.725, 1.267]
Abnormal total cholesterol (>5.18 mmol/L)	150(37.97%)	238(34.77%)	0.298	0.871 [0.670, 1.131]
Abnormal high density lipoprotein (<1.04 mmol/L)	27(6.84%)	70(10.21%)	0.065	1.549 [0.970, 2.475]
Abnormal low density lipoprotein (>3.19 mmol/L)	110(27.85%)	213(31.10%)	0.269	1.170 [0.886, 1.544]

***Correlation is significant at the 0.001 level (2-tailed).

A high fasting glucose level (>5.6 mmol/L) in addition to dyslipidaemia values of triglyceride (>1.70 mmol/L), total cholesterol (>5.18 mmol/L), high density lipoprotein (HDL) (<1.04 mmol/L), and low-density lipoprotein (LDL) (>3.19 mmol/L) are indicative of hyperglycaemia, according to the clinical indexes. A comparison of the prevalence of hyperglycaemia and dyslipidaemia in both groups revealed that the occurrence of hyperglycaemia was higher among the psoriatic patients than the control group (20.74% compared with 8.35%, *P* <0.001). However, the lipid profile showed no difference between the cases and the controls (*P* > 0.05) (Table 1).

### Blood Glucose and Lipid Levels in Patients With Psoriasis Pre- and Post-acitretin Treatment

**Figure 1:**
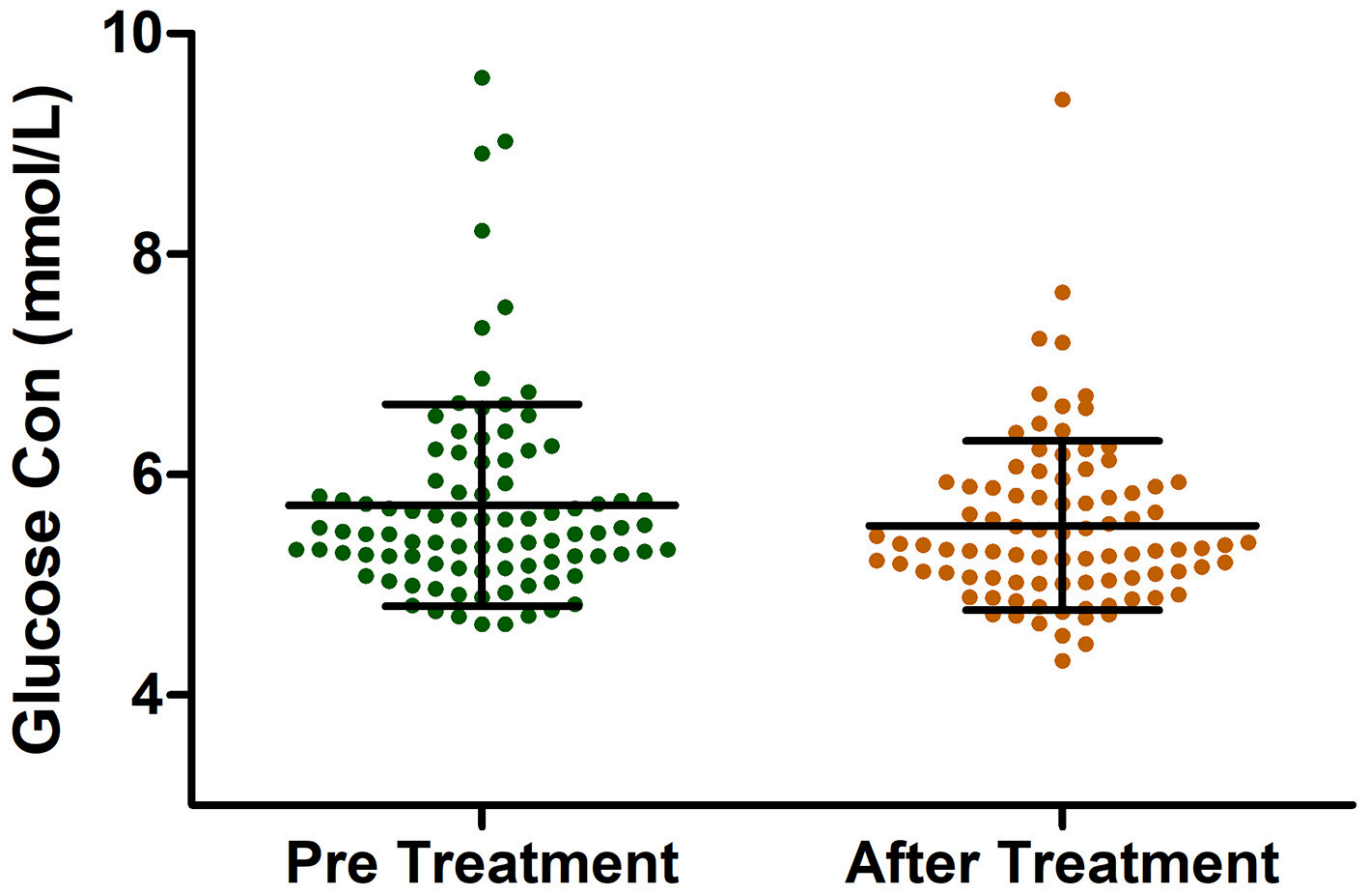
The Glucose Levels in patients with psoriasis pre and after acitretin treatment for 8 weeks.

Among the enrolled patients, 247 patients received acitretin therapy, and their BMI values and lipid profiles were recorded. We compared each patient's BMI value, blood lipid level, and other indicators pre- and post-acitretin treatment using a paired *t*-test and found that several parameters changed significantly. After receiving acitretin therapy for 8 weeks, the triglyceride and LDL levels increased (*P* < 0.05) while the HDL levels and BMI values significantly decreased (*P* < 0.05), as shown in Table 2. There was no observed significant difference in the patients' lipids levels at 12 weeks (*N* = 37) and 16 weeks (*N* = 10) (Supplementary Table 3). In this retrospective study, 95 of 247 patients with psoriasis who were treated with acitretin had their blood glucose levels checked before and after treatment. Interestingly, the result showed that the glucose level of the patients significantly decreased after acitretin treatment for 8 weeks (5.71 ± 0.91 vs. 5.55 ± 0.76 mmol/L, *P* = 0.0019) (Figure 1). For the fasting blood glucose level at 12 weeks was recorded in only 5 patients, therefore it was not suitable to explore the blood glucose change in such a small sample size.

**Table 2:** Fasting blood glucose and lipids levels change in patients with psoriasis after acitretin treatment for 8 weeks.

**Laboratory parameters**	**Pre-treatment** **(*N* = 247)**	**Post-treatment (*N* = 247)**	**Sig**.
BMI (kg/m^2^)	23.38 ± 0.31	23.22 ± 0.30	0.040*
ALT (U/L)	25.11 ± 14.40	29.76 ± 27.58	0.002**
AST (U/L)	25.05 ± 11.45	28.44 ± 18.79	<0.001***
BUN (mmol/L)	4.76 ± 2.79	4.48 ± 1.36	0.213
Creatinine (μmol/L)	92.17 ± 49.74	86.67 ± 18.50	0.179
Plasma uric acid (μmol/L)	337.41 ± 89.99	336.03 ± 86.45	0.807
Total cholesterol (TC) (mmol/L)	4.84 ± 1.01	4.91 ± 1.02	0.118
Triglycerides (TG) (mmol/L)	1.47 ± 0.87	1.70 ± 0.97	<0.001***
High density lipoprotein (HDL) (mmol/L)	1.39 ± 0.34	1.33 ± 0.30	<0.001***
Low density lipoprotein (LDL) (mmol/L)	2.81 ± 0.82	2.89 ± 0.79	0.027*
Blood glucose (mmol/L) (*N* = 95)	5.71 ± 0.91	5.55 ± 0.76	0.0019**

***Correlation is significant at the 0.001 level (2-tailed); ^*^Correlation is significant at the 0.05 level (2-tailed); **Correlation is significant at the 0.01 level (2-tailed).

### Uptake of 2-NBDG Significantly Increased With Acitretin Incubation

**Figure 2:**
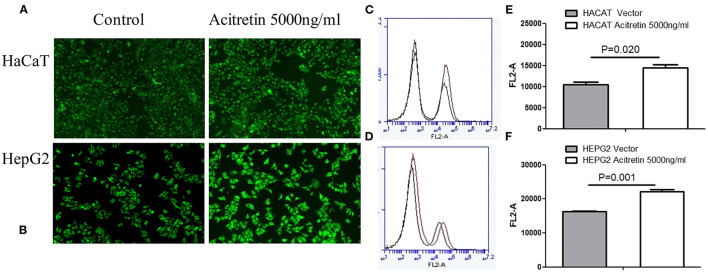
The uptake rate of 2-NBDG in HaCat cells and HepG2 cells when treated with acitretin. Legend: **(A,B)** The uptake of 2-NBDG by HaCaT and HepG2 cells was observed by fluorescence microscopy with or without acitretin incubation for 36h; **(C,D)** The uptake of 2-NBDG by HaCaT cells was measured by flow cytometry with or without acitretin incubation for 36h; **(D,F)** The uptake of 2-NBDG by HepG2 cells was measured by flow cytometry with or without acitretin incubation for 36h.

Fluorescence microscopy and flow cytometry were used to observe the uptake of 2-NBDG. As shown in Figure 2, the HepG2 and HaCaT cells treated with acitretin (5000 ng/ml), had an increased uptake of 2-NBDG compared to the control cells (*P* < 0.05).

### Increased Expression of Several Genes After Acitretin Incubation

**Figure 3:**
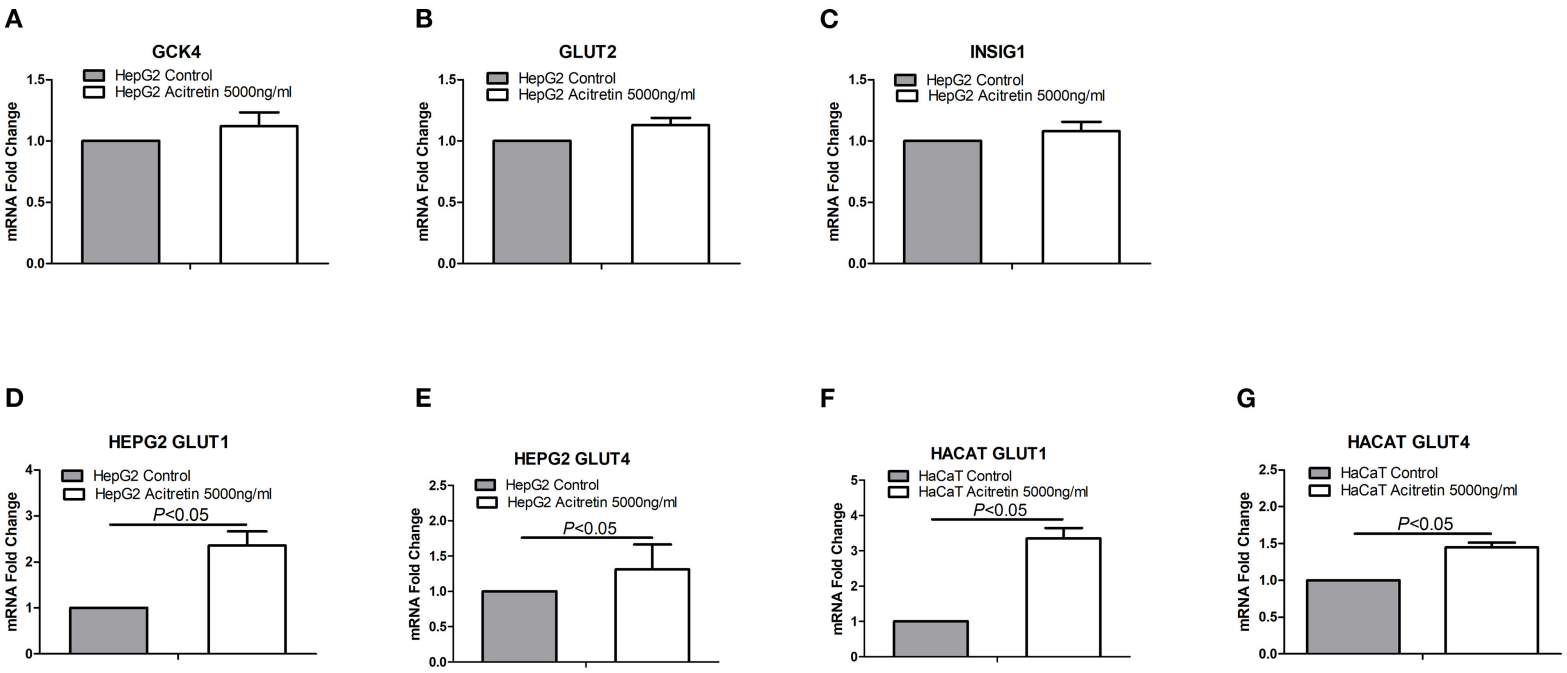
The expression of genes in HaCaT and HepG2 cells when treated with acitretin. Legend: **(A–C)** the transcript level expression of GCK4, GLUT2 and INSIG1 genes in HepG2 cells with or without the incubation of acitretin 5000ng/ml; **(D,E)** the GLUT1 and GLUT4 genes expression in HepG2 cells with or without acitretin incubation; **(F,G)** the GLUT1 and GLUT4 genes expression in HaCaT cells with or without acitretin incubation.

The transcript-level expression of both *GLUT1* and *GLUT4*, which are associated with glucose uptake, transport, and metabolism, were significantly increased in both HepG2 and HaCaT cells after acitretin incubation. However, expression of the related genes, *GLUT2, GCK4*, and *INSIG1*, was not significantly different as shown in Figure 3.

## Discussion

In this study, we confirmed that patients with psoriasis had a higher blood glucose level than those without (*P* < 0.001). Therefore, reducing hyperglycaemia is an important issue for psoriatic patients. Interestingly, the study revealed that the blood glucose level significantly decreased after acitretin treatment. In addition to a remarkable decrease in fasting blood glucose levels, patients treated with acitretin also had a reduction in their BMI. This suggests that acitretin may be a potential therapeutic for psoriatic patients of high glucose level or high BMI value.

Acitretin is widely used in Chinese psoriatic patients due to its low cost and mild side-effects. Previous studies pointed out that both the basal serum glucose level (*P* < 0.001) and the body weight index (*P* < 0.05) decreased in ob/ob mice after all-trans retinoic acid (at-RA) was administered (18, 19). A possible rationale is that the retinol activation pathway is impaired in diabetes and obesity, and administering low-dose at-RA ameliorates adiposity, glucose intolerance, and insulin resistance (20). Corbetta et al. found that a low dosage of acitretin induced a mild, transient reduction of insulin sensitivity (21), which is consistent with our findings.

Several studies have shown that retinoids induce the secretion and sensitivity of insulin and gluconeogenesis, resulting in a reduction in the incidence of early obesity (22–28). This indicates that RA is an effective reference anti-diabetic agent (18, 26). While the detailed mechanisms by which RA affects glucose metabolism remain undiscovered, studies have pointed out that retinoids increase the expression of tyrosine protein phosphatase 1B (29) and enhance ATP synthesis in the presence of the PKCδ signalosome (30), or increase the ratio of serum retinol-binding protein 4 (26). Other studies have also indicated that retinoids potentially stimulate glucose uptake by activating p38 mitogen-activated protein kinase (31), retinoic acid receptors, retinoid X receptors, and PPAR gamma heterodimers (18, 32, 33). Additionally, retinoids restore the down-regulated expression of *GLUT4* (34). *GLUT4* is the main insulin-sensitive glucose transporter that facilitates tissue and cellular uptake of glucose (34). In this study, we found that acitretin upregulated *GLUT4* and *GLUT*1 expressions in HaCaT and HepG2 cells. This contributed to the cellular uptake of peripheral blood glucose, resulting in a decrease in the fasting blood glucose levels, confirming the antihyperglycemic effect of acitretin in Chinese psoriasis patients. It has been shown that glucose transport in keratinocytes is mediated largely by the *GLUT1* facilitative transporter. *GLUT1* inactivation or inhibitor-assisted decrease resulted in proliferative hyperplasia in mouse models with a psoriasis-like disease (35, 36). From this point of view, the mechanism of acitretin on psoriasis is independent of the inhibition of the glucose metabolic pathway. However, this claim needs to be validated in a large population and using *in vitro* studies.

Additionally, from the results, there was no observable difference in the lipid profile between psoriatic patients and the control group. Hyperlipidaemia is the most commonly reported ADR to acitretin. Elevated serum triglyceride levels have been reported in ~1.7–50% of patients (37, 38), hypercholesterolaemia in 10–30% of patients, and decreased HDL levels have been found in ~40% of patients taking acitretin (8). Several other studies have also suggested that long-term retinoid therapy may increase the risk of atherosclerosis (16). Our results revealed a significant change in the patients' lipid profile (elevated cholesterol, triglyceride, and LDL levels, and decreased HDL levels), which is consistent with previous studies. However, this change is gradual and is considered (or will become) insignificant with treatment over time. This claim is consistent with reports from Chularojanamontri et al. (15), who stated that long-term and low-dose acitretin treatment in patients with psoriasis is unlikely to cause liver or lipids abnormalities. It is speculated that there is a homeostasis between glucose and lipids in the body, and retinoids is thought to regulate this homeostasis (18). The more peripheral glucose is absorbed by tissues after retinoids treatment, then the less glucose is converted to triglycerides.

There are a few limitations associated with this study. First, the results require further validation using a larger sample size and a multicentre study. Second, the changes in blood glucose levels were observed in patients with short-term treatment in this retrospective study; therefore, it is necessary to extend the duration of acitretin treatment. Finally, the results were obtained experimentally by clinical observation, which needs to be further elucidated through elaborate molecular research.

## Conclusion

In this study, we confirmed that the blood glucose levels are elevated in Chinese psoriatic patients compared to those without psoriasis. We also demonstrated that acitretin exerts an apparent effect on lowering the blood glucose levels and the BMI values in Chinese patients with psoriasis. Additionally, it was observed that acitretin up-regulates the expression of *GLUT1* and *GLUT4* in HaCaT and HepG2 cells, potentially leading to a decrease in the peripheral blood glucose level. This indicates that acitretin may be used to control the blood glucose level in psoriatic patients, especially those with diabetes.

## Data Availability Statement

The original contributions presented in the study are included in the article/Supplementary Material, further inquiries can be directed to the corresponding author.

## Ethics Statement

The studies involving human participants were reviewed and approved by the Ethic Committee of Xiangya Hospital. The patients/participants provided their written informed consent to participate in this study. Written informed consent was obtained from the individual(s) for the publication of any potentially identifiable images or data included in this article.

## Author Contributions

WC and MC finished the data collection and operation. JS, CL, and WZ organized the paper. XC supported the funding. YK and HQ analyzed the data. All authors contributed to the article and approved the submitted version.

## Funding

This study was funded by grants received from the National Natural Science Foundation of China program (Nos. 81803118, 81903222, 81830096, and 81974479).

## Conflict of Interest

The authors declare that the research was conducted in the absence of any commercial or financial relationships that could be construed as a potential conflict of interest.

## Publisher's Note

All claims expressed in this article are solely those of the authors and do not necessarily represent those of their affiliated organizations, or those of the publisher, the editors and the reviewers. Any product that may be evaluated in this article, or claim that may be made by its manufacturer, is not guaranteed or endorsed by the publisher.
