# Supplementary material for: Reductive Effect of Acitretin on Blood Glucose Levels in Chinese Patients With Psoriasis
Source: Front Med (Lausanne). 2021 Dec 16;8:764216. doi: 10.3389/fmed.2021.764216 (PMC8716687; doi:10.3389/fmed.2021.764216)
Supplement: Supplementary file 1 [file Data_Sheet_1.doc]

Supplement Table 1. The primers for genes RT-PCR
Gene	Forward Primer	Reverse Primer	
GLUT1	TCACTGTCGTGTCGCTGTTT	ACGATGAACCATGGGATGGG	
GLUT2	TGACAGGACTCCCAACTAGTACAAT	AGGCCTGAAATTAGCCCTGT	
GLUT4	AGAGCCAGCTCTCTCTACCC	GTCACACGAGGGGAATGAGG	
GCK4	CCTTCTCCGCACACAAATGC	CCGCGTTTGACATGTTCACTG	
INSIG1	CTCTCGGCCAGGAAGCG	GATGCCAGGTGTTGGGGTAG	


Supplement Table 2. The other laboratory tests parameters between psoriasis and controls groups
Laboratory tests parameters	Controls(N=395)	Psoriasis(N=685)	Sig.	
Albumin (g/L)	48.21¡À4.02	46.68¡À3.38	<0.001***	
Globulin (g/L)	26.51¡À3.47	27.70¡À3.78	<0.001***	
Albumin/Globulin ratio	1.86¡À0.29	1.72¡À0.28	<0.001***	
Total bilirubin (¦Ìmol/L)
blood urea nitrogen (mmol/L)	12.35¡À7.42
5.11¡À1.98	11.40¡À4.98
4.72¡À1.35	0.013*
<0.001***	
Plasma uric acid (¦Ìmol/L)	328.28¡À85.11	337.52¡À85.87	0.101	
White blood cell count (10^9/L)
Neutrophil count(10^9/L)	6.4¡À1.7
3.62¡À1.36	7.23¡À1.9
4.5¡À1.59	<0.001***
<0.001***	
Lymphocyte count (10^9/L)	2.22¡À1.61	2.00¡À0.66	<0.001***	
Monocyte count (10^9/L)	0.45¡À0.15	0.53¡À0.19	<0.001***	
Neutrophil percentage (%)	55.73¡À8.22	30.34¡À30.62	<0.001***	
Lymphocyte percentage (%)	34.3¡À7.62	14.73¡À15.68	<0.001***	
Monocyte percentage (%)
mean corpuscular hemoglobin concentration(MCHC)	6.95¡À1.75
337.19¡À9.71	3.66¡À3.86
333.18¡À15.58	<0.001***
<0.001***	
***. Correlation is significant at the 0.001 level (2-tailed); **. Correlation is significant at the 0.01 level (2-tailed); *. Correlation is significant at the 0.05 level (2-tailed).
Table 1 legends: When compared with the controls, the psoriatic patients showed a statistical difference on some parameters, including albumin, globulin, albumin/globulin ratio, total bilirubin, blood urea nitrogen ,creatinine, blood Glucose, low density lipoprotein, white blood cell count, neutrophil percentage, lymphocyte percentage, monocyte percentage, and red blood cell distribution width.


Supplement Table 3. The lipids parameters levels in patients with psoriasis before and after the acitretin treatment for long treatment
Laboratory parameters	Pre-Treatment 0W vs. After-Treatment 8W (N=37)	 Sig.	Pre-Treatment 0W vs. After-Treatment 12W (N=37) 	 Sig.	Pre-Treatment 0W vs. After-Treatment 16W (N=10)	 Sig.	
Total Cholesterol (TC)	4.94¡À1.15 vs.5.15¡À1.00 	0.152	4.94¡À1.15 vs.4.97¡À0.97 	0.821	5.15¡À1.15 vs.4.97¡À0.70	0.685	
Triglycerides (TG) (mmol/L)	1.53¡À0.79 vs.1.83¡À0.80	0.020*	1.53¡À0.79 vs.1.72¡À0.86	0.225	1.40¡À0.57 vs.1.58¡À0.43	0.233	
High density lipoprotein (HDL)	1.30¡À0.34 vs.1.30¡À0.28	0.762	1.30¡À0.34 vs. 1.24¡À0.31	0.100	1.39¡À0.44 vs. 1.39¡À0.38	0.966	
Low density lipoprotein (LDL)	3.05¡À0.77 vs.3.17¡À0.74	0.036*	3.05¡À0.77 vs. 3.12¡À0.67	0.331	3.04¡À0.75 vs.2.99¡À0.43	0.768	


 
