# Supplementary material for: Reductive Effect of Acitretin on Blood Glucose Levels in Chinese Patients With Psoriasis
Source: Front Med (Lausanne). 2021 Dec 16;8:764216. doi: 10.3389/fmed.2021.764216 (PMC8716687; doi:10.3389/fmed.2021.764216)
Supplement: Supplementary file 2 [file Data_Sheet_2.docx]

The Raw Data of the paper “Reductive Effect of Acitretin on Blood Glucose Levels in Chinese Patients with Psoriasis” was updated on the website:

https://www.jianguoyun.com/p/DbafX5cQipnrCRj59owE
